# Identification of Key MicroRNAs and Mechanisms in Prostate Cancer Evolution Based on Biomarker Prioritization Model and Carcinogenic Survey

**DOI:** 10.3389/fgene.2020.596826

**Published:** 2021-01-15

**Authors:** Yuxin Lin, Zhijun Miao, Xuefeng Zhang, Xuedong Wei, Jianquan Hou, Yuhua Huang, Bairong Shen

**Affiliations:** ^1^Department of Urology, The First Affiliated Hospital of Soochow University, Suzhou, China; ^2^Department of Urology, Suzhou Dushuhu Public Hospital, Suzhou, China; ^3^Institutes for Systems Genetics, Frontiers Science Center for Disease-Related Molecular Network, West China Hospital, Sichuan University, Chengdu, China

**Keywords:** miRNA biomarker, prostate cancer management, miRNA-mRNA network modeling, miRNA regulatory pattern, systems biology

## Abstract

**Background:** Prostate cancer (PCa) is occurred with increasing incidence and heterogeneous pathogenesis. Although clinical strategies are accumulated for PCa prevention, there is still a lack of sensitive biomarkers for the holistic management in PCa occurrence and progression. Based on systems biology and artificial intelligence, translational informatics provides new perspectives for PCa biomarker prioritization and carcinogenic survey.

**Methods:** In this study, gene expression and miRNA-mRNA association data were integrated to construct conditional networks specific to PCa occurrence and progression, respectively. Based on network modeling, hub miRNAs with significantly strong single-line regulatory power were topologically identified and those shared by the condition-specific network systems were chosen as candidate biomarkers for computational validation and functional enrichment analysis.

**Results:** Nine miRNAs, i.e., *hsa-miR-1-3p, hsa-miR-125b-5p, hsa-miR-145-5p, hsa-miR-182-5p, hsa-miR-198, hsa-miR-22-3p, hsa-miR-24-3p, hsa-miR-34a-5p*, and *hsa-miR-499a-5p*, were prioritized as key players for PCa management. Most of these miRNAs achieved high AUC values (AUC > 0.70) in differentiating different prostate samples. Among them, seven of the miRNAs have been previously reported as PCa biomarkers, which indicated the performance of the proposed model. The remaining *hsa-miR-22-3p* and *hsa-miR-499a-5p* could serve as novel candidates for PCa predicting and monitoring. In particular, key miRNA-mRNA regulations were extracted for pathogenetic understanding. Here *hsa-miR-145-5p* was selected as the case and *hsa-miR-145-5p/NDRG2/AR* and *hsa-miR-145-5p/KLF5/AR* axis were found to be putative mechanisms during PCa evolution. In addition, *Wnt* signaling, prostate cancer, microRNAs in cancer etc. were significantly enriched by the identified miRNAs-mRNAs, demonstrating the functional role of the identified miRNAs in PCa genesis.

**Conclusion:** Biomarker miRNAs together with the associated miRNA-mRNA relations were computationally identified and analyzed for PCa management and carcinogenic deciphering. Further experimental and clinical validations using low-throughput techniques and human samples are expected for future translational studies.

## Introduction

Prostate cancer (PCa) is a kind of malignant tumors which ranks first in the incidence of male leading cancer types according to the reports from Cancer Statistics 2020 (Siegel et al., 2020). It has been acknowledged that the occurrence and progression of PCa are highly heterogeneous, resulting in the difficulty in PCa precision medicine and personalized healthcare. In clinical practice, although the level of serum prostate-specific antigen (*PSA*) and multi parameter Magnetic Resonance Imaging (MRI) techniques are widely tested for PCa screening, the sensitivity and specificity are still need to be measured for improving positive detection rate and avoiding unnecessary biopsy.

As a class of post-transcriptional regulators, microRNAs (miRNAs) are found to be active in carcinogenesis, including PCa (Khanmi et al., 2015). Extensive efforts showed that miRNAs could regulate down-stream messenger RNAs (mRNAs) though complementary base pairing and eventually affect the signal transmission of pathways and the function of cellular activities (Esteller, 2011). Currently, the identification and prioritization of miRNAs as biomarkers for PCa theranostics is of clinical interest, which would help the early diagnosis, prognosis tracking and targeted therapy of PCa patients (Bhagirath et al., 2018; Wei et al., 2020).

In the era of artificial intelligence and biomedical informatics, data-driven translational PCa research brings a new frontier for systems modeling of complex genetic interactions (Lin et al., 2020). The structural characteristics within biological networks offer great opportunities for understanding cancer heterogeneity at systems biology level (Liu Y. Y. et al., 2011). Accumulating evidences have demonstrated the functional importance of hub nodes in gene network for prioritizing key players during PCa development. For example, Zhu et al. identified five miRNAs and seven genes for predicting the biochemical recurrence-free survival of PCa by evaluating the significance of differential expression and the location of genes in the network (Zhu et al., 2020). Analogously, Tu et al. proposed an integrated framework that considers both dynamical changes of gene expression and static features in protein-protein network for extracting key miRNA-mRNA pairs associated with docetaxel resistance in PCa (Tu et al., 2019).

It is reasonable that hub genes are located in the center of the network by directly connect more partner genes to control the information flow. In addition to such regulatory pattern, special structures hidden in the network are still worth being explored for investigating the strength of genes in network stability. Biomarkers hold the power to indicate the dynamical alternations in biological systems, searching for vulnerable hallmarks from miRNA-gene regulations would therefore provide crucial clues for cancer biomarker discovery (Lin et al., 2019). In our previous studies, we found that a certain number of genes were independently regulated by single miRNAs and a new parameter, i.e., NSR (number of single-line regulation) was defined based on network vulnerability theory to quantify the single-line regulation of miRNAs in miRNA-mRNA network. According to statistical evidences, miRNAs with higher NSR values were structurally important to serve as candidate biomarkers for cancer management (Lin et al., 2018b), and five miRNAs were computationally screened and validated for PCa metastasis (Lin et al., 2018a).

**Figure 1:**
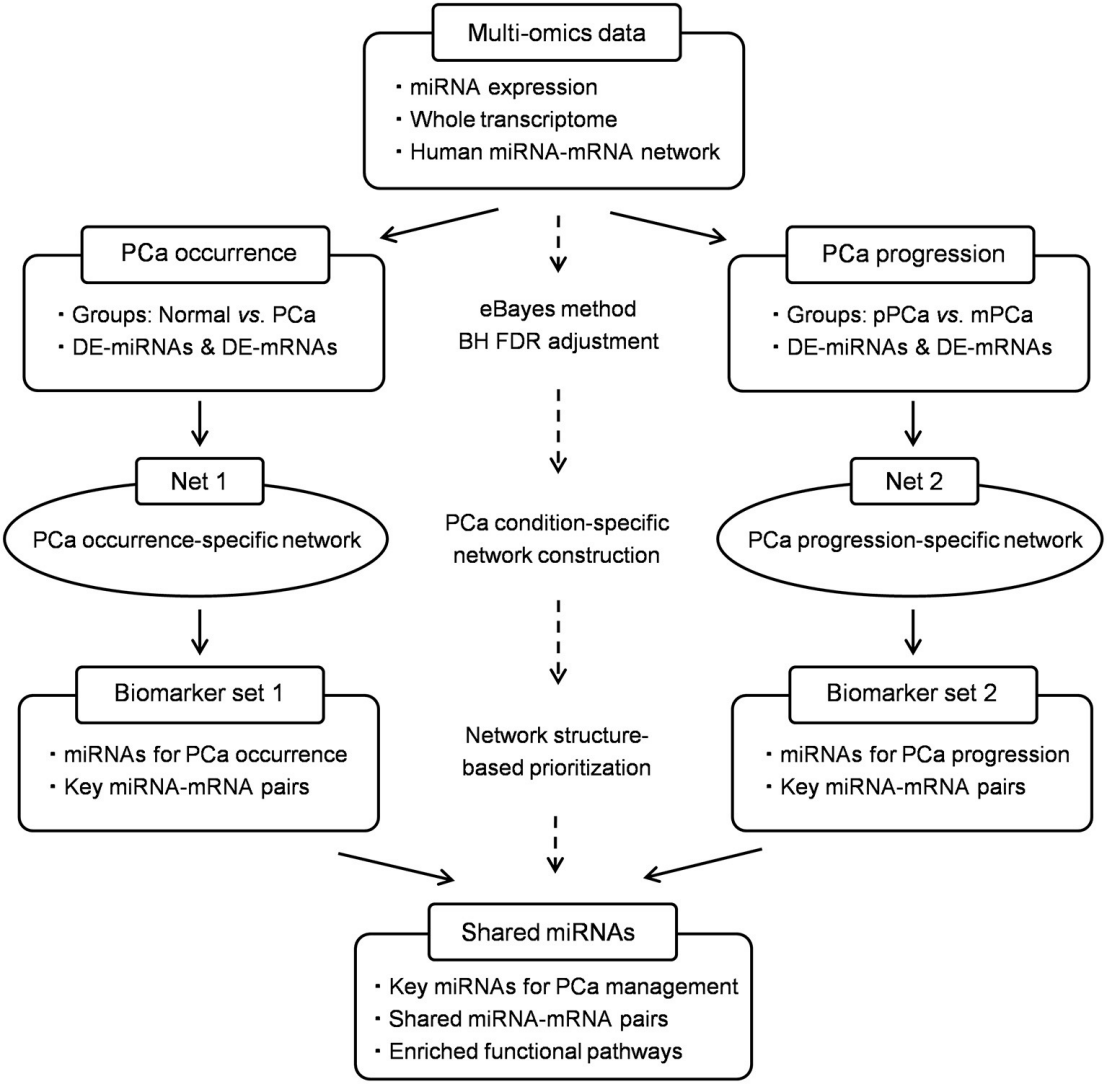
The schematic pipeline of this study. PCa, prostate cancer; pPCa, clinically localized primary prostate cancer; mPCa, metastatic prostate cancer; DE, differentially expressed; eBayes, empirical bayes; BH FDR, Benjamini-Hochberg false discovery rate.

On the basis of our previous findings, in this study we expand our research interest and update the bioinformatics framework to identify key miRNAs functionally important in the whole process during PCa evolution as both of the diagnosis and metastasis monitoring are hot topics for precision medicine. In methodology, two PCa condition-specific miRNA-mRNA networks, i.e., PCa occurrence and progression-specific network, are respectively constructed and characterized based on the integration of novel gene expression and network topological signatures. Meanwhile potential miRNA-mRNA pairs in PCa evolution are deciphered for functional survey and multi-level carcinogenesis understanding. In particular, the traditional hub property is improved by combing and measuring the single-line regulatory power of miRNAs in the computational simulation process, which would enhance the overall predictive performance and biological significance of the bioinformatics model. The schematic pipeline is shown in Figure 1.

## Materials and Methods

### Dataset Collection and Processing

The miRNA and mRNA datasets were both collected from gene expression omnibus (GEO) (Edgar et al., 2002), where the super-series GSE21032 provides the integrative genomic profiling of human PCa (Taylor et al., 2010), including the clinically localized primary PCa (pPCa), metastatic PCa (mPCa) and the normal adjacent benign prostate samples. Here the normalized datasets of sub-series GSE21036 and GSE21034 were downloaded for further analysis. As illustrated in Table 1, GSE21036 contains a total of 99 pPCa, 14 mPCa and 28 normal miRNA samples screened by Agilent-019118 Human microRNA Microarray 2.0 G4470B platform, whereas GSE21034 consists of the whole-transcript expression data for 131 pPCa, 19 mPCa, and 29 normal control prostate samples profiled on Affymetrix Human Exon 1.0 ST Array. In addition, GSE54516 with miRNA expression data measured in prostate benign and tumor tissues using miRNA Taqman plates was chosen as an independent dataset for result validation (Gu et al., 2015).

**Table 1:** Datasets used in this study.

**RNA type**	**GEO accession**	**Platform**	**Sample source**	**Normal sample**	**PCa sample**	
					**pPCa**	**mPCa**
miRNA	GSE21036	GPL8227	Human prostate	28	99	14
mRNA	GSE21034	GPL10264	Human prostate	29	131	19
miRNA	GSE54516	GPL18234	Human prostate	48	51	

*PCa, prostate cancer; pPCa, clinically localized primary prostate cancer; mPCa, metastatic prostate cancer; GEO, Gene Expression Omnibus*.

To ensure the specificity of the RNAs in PCa genesis, differential expression analysis was performed and compared among different sample groups, i.e., normal vs. PCa and pPCa vs. mPCa. Based on the evaluation of statistics approaches for generating differentially expressed (DE) genes from Microarray data (Jeffery et al., 2006), the empirical bayes (eBayes) method was chosen for raw *p*-value calculation (Smyth, 2004) and the Benjamini-Hochberg false discovery rate (FDR) was then applied to adjust raw *p*-values. For the gene associated with multiple probes, the probe with the most significant variation was selected and assigned. The criterion for DE-miRNA and DE-mRNA identification was defined as the adjusted *p*-value (adj. *p*-value) < 0.05.

### Model Development Based on Network Construction and Characterization

The bioinformatics model was developed based on the characterization of miRNA regulation in PCa condition-specific miRNA-mRNA network. As shown in Figure 1, a human global miRNA-mRNA network was first constructed as the reference by integrating both experimentally validated and computationally predicted miRNA-mRNA pairs from public databases and software tools (Lin et al., 2018b). Then DE-miRNAs and DE-mRNAs were mapped onto the given network to extract PCa condition-specific networks. In this study a total of two networks, i.e., PCa occurrence-specific and progression-specific networks, were measured, respectively, where the former described the role of miRNAs in PCa occurrence process and the latter simulated miRNA regulation during PCa progression and metastasis.

To quantity the regulatory pattern of miRNAs in the network, the feature parameters NTG (number of targeted genes) and NSR were defined and used for biomarker prioritization. Among them, NTG represents the number of genes targeted by certain miRNAs. According to the theory of network sciences, hub nodes with more links in the network are functionally important in biological systems. Meanwhile our previous findings have demonstrated that biomarker miRNAs held strong single-line regulatory power in the network since the single-line points are vulnerable and the dysregulation in such sites are likely to cause the disorder at the systems level (Lin et al., 2018b, 2019). Thus, NSR parameter is set to indicate the number of genes independently regulated by a given miRNA. In addition, the ratio NSR/NTG was calculated to further evaluate the significance of miRNAs on gene regulation.

Based on above network systems and feature parameters, miRNAs with significantly high NTG, NSR, and NSR/NTG values (*p*-value < 0.05, Wilcoxon signed-rank test) were prioritized in each PCa-specific network and those shared by the two networks were selected as key players for predicting the occurrence and progression of PCa. Moreover, the shared miRNA-mRNA pairs were also identified for functional and carcinogenic survey.

### Performance Evaluation and Comparison

The receiver-operating characteristic (ROC) and clustering analysis were performed based on the expression data of the identified miRNAs using ROCR and Pheatmap package in R program, respectively. Here the area under ROC curve (AUC) was calculated for each miRNA to evaluate and compare the biomarker potential on differentiating prostate samples, i.e., normal vs. PCa and pPCa vs. mPCa. Moreover, an additional index called prediction precision was defined as the percentage of literature-reported PCa miRNA biomarkers in the whole predicted set to validate and compare the performance of the proposed model.

### Functional Exploration and Carcinogenic Analysis

The functional carcinogenesis of the identified miRNAs in PCa evolution was investigated based on the research paradigm of miRNA-gene-pathway axis. First the targets of miRNAs were retrieved from each PCa condition-specific network and miRNAs-mRNAs shared by the two networks were then collected as key regulations for gene ontology (GO) annotation and Kyoto Encyclopedia of Genes and Genomes (KEGG) pathway enrichment analysis using the online tool Database for Annotation, Visualization and Integrated Discovery (DAVID, version 6.8) (Kanehisa and Goto, 2000; Huang da et al., 2009). The top ten significant terms with *p*-value < 0.05 were chosen for pathogenic understanding of their associations with cellular proliferation, invasion, metastasis and the responses to PCa treatment through literature exploration.

## Results

### Biomarker miRNAs Identified for PCa Management

In this study, two PCa condition-specific networks, i.e., occurrence-specific and progression-specific network, were respectively, extracted based on human miRNA-mRNA reference network and the selected sample datasets. Among them, the occurrence-specific network comprised 6,063 regulatory pairs associated with 138 DE-miRNAs and 2,035 DE-mRNAs between normal and PCa samples. In the progression-specific network, a total of 7,510 regulations among 169 DE-miRNAs and 2,238 DE-mRNAs with the expression change in PCa progression and metastasis were statistically identified.

After network structure-based filtration, 17 and 19 miRNAs with significantly high NTG, NSR and NSR/NTG values were computationally screened in PCa occurrence-specific and progression-specific network, respectively (see Supplementary Table 1), and the shared nine miRNAs, i.e., *hsa-miR-1-3p, hsa-miR-125b-5p, hsa-miR-145-5p, hsa-miR-182-5p, hsa-miR-198, hsa-miR-22-3p, hsa-miR-24-3p, hsa-miR-34a-5p*, and *hsa-miR-499a-5p*, were finally collected as key players during PCa evolution. As illustrated in Table 2, *hsa-miR-182-5p* and *hsa-miR-198* were over-expressed in the initiation and metastasis processes of PCa, whereas the remaining seven miRNAs were down-regulated during PCa development.

**Table 2:** Statistics and topological features of the identified miRNAs.

**miRNA**	**Expression**	**adj**. ***p*****-value**		**NTG**		**NSR**		**NSR/NTG**	
		**I**	**II**	**I**	**II**	**I**	**II**	**I**	**II**
*hsa-miR-1-3p*	Down	4.10e-3	1.10e-30	69	75	15	17	0.2174	0.2267
*hsa-miR-125b-5p*	Down	3.03e-3	8.98e-16	45	48	12	8	0.2667	0.1667
*hsa-miR-145-5p*	Down	1.96e-7	8.00e-25	56	57	11	17	0.1964	0.2982
*hsa-miR-182-5p*	Up	1.25e-9	2.46e-2	47	44	10	7	0.2128	0.1591
*hsa-miR-198*	Up	9.19e-3	7.67e-5	50	47	14	9	0.28	0.1915
*hsa-miR-22-3p*	Down	4.11e-3	2.45e-2	83	82	19	14	0.2289	0.1707
*hsa-miR-24-3p*	Down	5.82e-6	1.12e-9	39	43	7	10	0.1795	0.2326
*hsa-miR-34a-5p*	Down	1.20e-2	2.24e-2	58	59	9	14	0.1552	0.2373
*hsa-miR-499a-5p*	Down	4.22e-4	3.13e-2	73	76	10	10	0.1370	0.1316

*adj.p-value, adjusted p-value; NTG, number of targeted genes; NSR, number of single-line regulation; I, Normal vs. PCa; II, pPCa vs. mPCa*.

**Figure 2:**
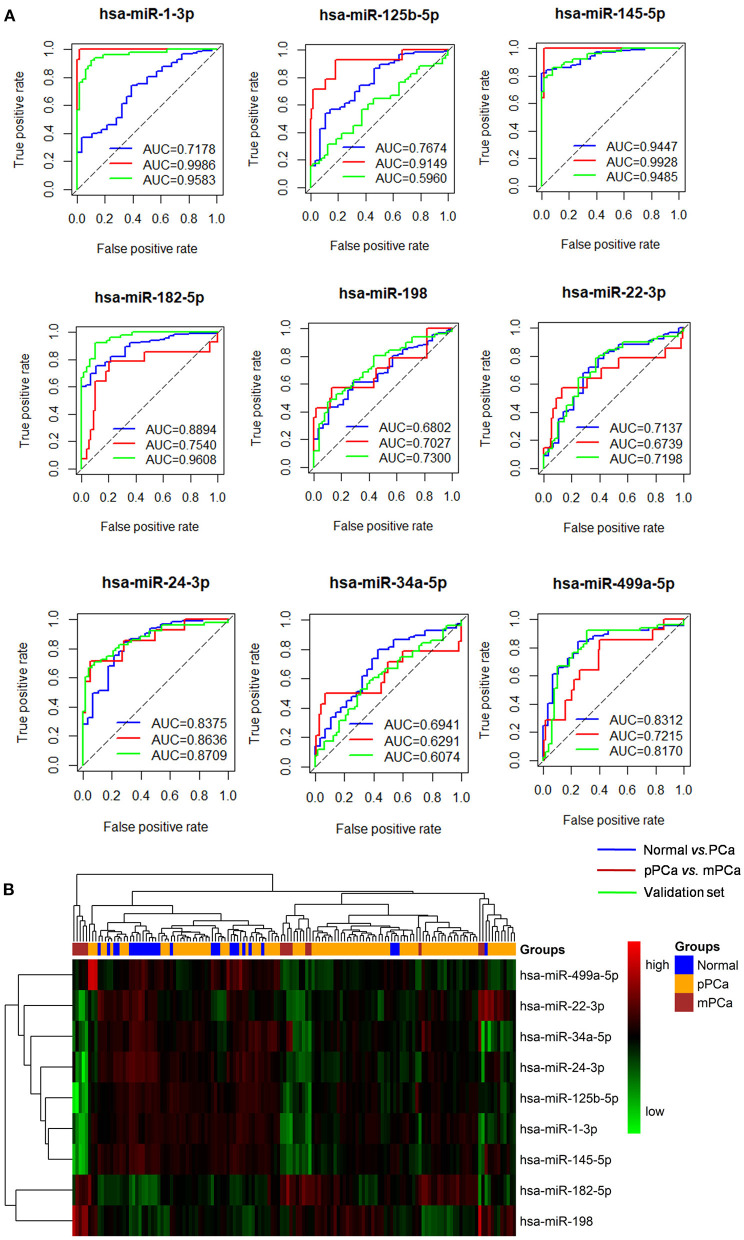
The ROC and clustering analysis for the identified miRNAs. **(A)** ROC analysis. Blue curve: Normal vs. PCa for diagnostic performance evaluation; Red curve: pPCa vs. mPCa for prognostic and subtyping performance evaluation. Green curve: validation based on an independent dataset. **(B)** Clustering analysis. The blue, orange and red blocks represent normal, pPCa and PCa sample, respectively, and the degree of green and red in the map indicates the relative expression level of miRNAs from low to high, respectively. ROC, the receiver-operating characteristic curve; AUC, area under the ROC curve; PCa, prostate cancer; pPCa, clinically localized primary prostate cancer; mPCa, metastatic prostate cancer.

As shown in Figure 2A, the ROC analysis strengthened the power of the identified miRNAs for classifying different prostate samples, i.e., normal vs. PCa and pPCa vs. mPCa. For example, the average AUC between the groups of normal and PCa was 0.7862 (ranged from 0.6802 to 0.9447), and it reached 0.8057 (ranged from 0.6291 to 0.9986) for discriminating pPCa and mPCa samples. In the validation set, the average AUC was 0.8010 (ranged from 0.5960 to 0.9608), which was comparable with that in the prediction set. In particular, *hsa-miR-145-5p* achieved the overall best performance on PCa predicting and subtyping (both AUC > 0.9), demonstrating its prospects for carcinogenic study and future clinical translation. However, as shown in Figure 2B the three groups were not well distinguished by combing the expression signature of these miRNAs, which indicated that the identified miRNAs would not be suitable to serve as potential biomarker combinations.

### Literature-Based Functional Annotation and Validation

According to the review of citations in PubMed, seven of the identified miRNAs (77.8%, 7/9), i.e., *hsa-miR-1-3p, hsa-miR-125b-5p, hsa-miR-145-5p, hsa-miR-182-5p, hsa-miR-198, hsa-miR-24-3p*, and *hsa-miR-34a-5p* have been previously reported as biomarkers or molecular tools for PCa prediction. For example, Xie et al., investigated the diagnostic value and carcinogenic mechanisms of *hsa-miR-1* (namely *hsa-miR-1-3p*) in PCa. The result of meta-analyses and bioinformatics studies showed that this miRNA was significantly down-expressed in PCa samples and it could regulate pathways associated with androgen receptor (*AR*) activities in PCa development (Xie et al., 2018). Hudson et al. performed *in vitro* analysis and proved the tumor suppressor function of *hsa-miR-1* in PCa cell proliferation and motility. Besides the down-regulation in pPCa samples, the expression of this miRNAs was found to be reduced in distant metastasis, which indicated its power for prediction PCa progression and recurrence (Hudson et al., 2012). Zhu et al. identified *hsa-miR-125b* (namely *hsa-miR-125b-5p*) as an independent factor indicating castration resistant in PCa (Zhu et al., 2015), and this miRNA could improve the prediction of PCa status on the basis of serum *PSA* screening (Roberts et al., 2015). Xu et al. evaluated the level of *hsa-miR-145* expression (namely *hsa-miR-145-5p*) in urinary extracellular vesicles between PCa and healthy or benign prostate hyperplasia subjects. They found that the expression of this miRNAs was significantly altered in the urine of PCa patients, which highlighted its potential for PCa non-invasive diagnosis (Xu et al., 2017). Moreover, this miRNA was both detected in our previous studies using different network systems and computational models, demonstrating its significance on tumor regulation in PCa invasion, metastasis, and castration resistance (Zhu et al., 2015; Lin et al., 2018a). Based on RT-qPCR testing and validation, Bidarra et al. reported that the level of *hsa-miR-182-5p* was related to the advanced stage of PCa pathogenesis, and it was over-expressed in the plasma samples of patients with metastasis (Bidarra et al., 2019). Similarly, the expression of *hsa-miR-198* was found to be increased especially in the cohorts of high-grade (Gleason score ≥ 8) PCa (Walter et al., 2013), which was consistent with the result in this study. In addition, *hsa-miR-24-3p* and *hsa-miR-34a-5p* were also functional regulators in progression and therapeutic intervention by targeting PCa-related genes. Lynch et al. used the PCR to investigate the role of *hsa-miR-24* (namely *hsa-miR-24-3p*) in PCa cell lines. Compared with the normal prostate epithelial cell line, *hsa-miR-24* was down-regulated in PCa. This pattern was closely correlated with higher level of serum *PSA* and other clinical indices for PCa monitoring. Moreover, *p27* (*CDKN1B*) and *p16* (*CDK2NA*) were confirmed as targets of this miRNA in PCa cells (Lynch et al., 2016). Liu et al. announced that *hsa-miR-34a* (namely *hsa-miR-34a-5p*) was a tumor suppressor gene and it could inhibit the stem cells and metastasis by directly targeting *CD44* (Liu C. et al., 2011). Meanwhile this miRNA was a predictive biomarker for docetaxel responses associated with PCa therapy (Corcoran et al., 2014).

Although the remaining miRNAs, i.e., *hsa-miR-22-3p* and *hsa-miR-499a-5p*, have not been reported as PCa biomarkers yet, they were also powerful for PCa classifying and subtyping based on ROC analysis of this study. Hence these two miRNAs could serve as novel candidates for PCa diagnosis and prognosis. In summary, in this study the proposed bioinformatics model outperformed our previous method by increasing the prediction precision from 40 to 77.8% (Lin et al., 2018a), and more experimental validations using wet-lab approaches are needed in the future work.

### Key miRNA-mRNA Regulations in PCa Carcinogenesis: A Focused Study on *hsa-miR-145-5p*

**Figure 3:**
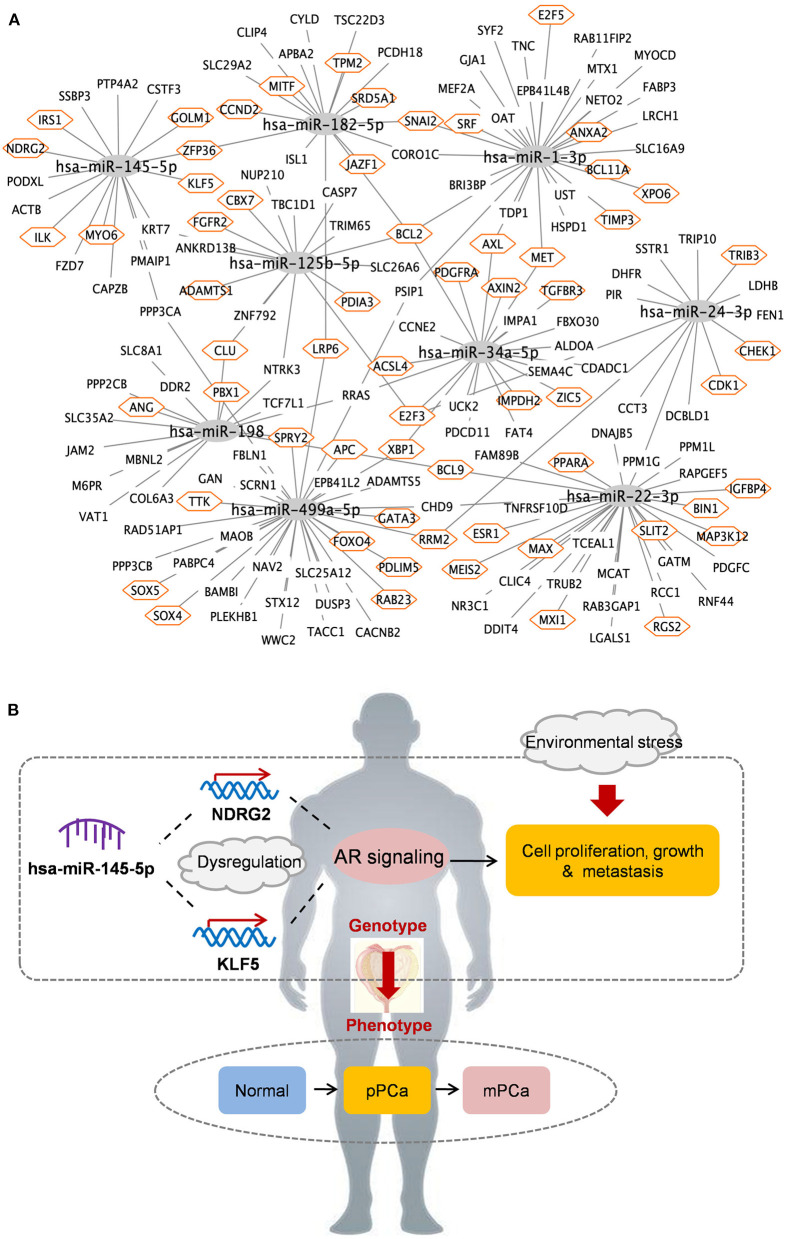
The identified miRNAs and key miRNA-mRNA regulations in PCa occurrence and progression. **(A)** Overview of regulatory associations. Orange hexagon: PCa-related genes. **(B)** Potential mechanisms of *hsa-miR-145-5p* in PCa evolution. pPCa, clinically localized primary prostate cancer; mPCa, metastatic prostate cancer.

A total of 194 miRNA-mRNA regulations among the nine miRNA biomarkers and 172 dysfunctional mRNAs were extracted from PCa occurrence and progression networks to investigate their carcinogenetic role in PCa evolution at the gene level. As shown in Figure 3A, approximately half of the mRNAs, single-line or co-regulated by the identified miRNA candidates, were involved in PCa genesis according to literature reports. In this study the regulations between *hsa-miR-145-5p* and known PCa-related genes were further analyzed since *hsa-miR-145-5p* was highly prioritized with the overall best performance on PCa prediction and subtyping in our ROC validation. As summarized in Table 3, *hsa-miR-145-5p* is a tumor suppressor in PCa development. It inhibited the *AR* signaling in PCa cells and the expression was inversely correlated with the change of *AR* and serum *PSA* level. In a well-characterized PCa cohort, this miRNA was found to be associated with the metastasis phenotype and could indicate the survival and the response to androgen deprivation therapy (Larne et al., 2015). Based on network extraction, seven PCa-related genes, i.e., *NDRG2, KLF5, IRS1, ZFP36, GOLM1, MYO6*, and *ILK*, were identified as potential targets in PCa carcinogenesis. Among them, *NDRG2* is a prognostic biomarker and negative regulator downstream of *AR* (Ren et al., 2014; Yu et al., 2015). It predicts PCa clinicopathologic features such as malignant and metastatic progression and affects the growth of androgen-dependent and castration-resistant PCa (Yu et al., 2015). Li et al. showed that *KLF5* was a key factor in androgen-*AR* signaling. It promoted the proliferation of PCa cells and could serve as a therapeutic target for PCa treatment (Li et al., 2020). As a famous star driving the carcinogenesis of PCa from normal prostate tissue to cancer biology, the *AR* signaling has already been explored across different researches, and the results in this study computationally demonstrated new insights and improved the understandings in *AR*-mediated PCa genesis. As described in Figure 3B, *hsa-miR-145-5p/NDRG2/AR* and *hsa-miR-145-5p/KLF5/AR* were inferred to be correlated with PCa evolution, which would be helpful for PCa diagnosis and therapy. In addition, *hsa-miR-145-5p* may regulate PCa cell proliferation, invasion, metastasis and apoptosis through other functional genes and associated pathways, which highlighted the underlying diversity and complexity in miRNA-PCa interaction (Persad et al., 2000; Neuhausen et al., 2005; Wang et al., 2016; Zhu et al., 2016; Yan et al., 2018).

**Table 3:** Functional mechanisms of *hsa-miR-145-5p* in PCa carcinogenesis.

**miRNA function**	**Potential target**	**Gene function**
• Suppressing *AR* signaling in PCa cells. • The level is inversely correlated with *PSA* alternation, the occurrence of metastasis phenotype and the response to androgen deprivation therapy. • PMID: 25969144	*NDRG2*	• A prognostic biomarker and regulator downstream of *AR*. • Associated with PCa malignant and metastatic progression. • Affecting the growth of androgen-dependent and castration-resistant PCa. • PMID: 24222185, 25756511
	*KLF5*	• A functional factor for *Androgen-AR* signaling. • Promoting cell proliferation in PCa cells. • PMID: 32245249
	*IRS1*	• G972R variant is associated with the risk of PCa occurrence. • PMID: 15678496
	*ZFP36*	• The expression change is associated with the overall survival and indicates the PCa biochemical recurrence. • PMID: 26563146
	*GOLM1*	• Promoting the progression of PCa via activating *PI3K-AKT-mTOR* signaling. • PMID: 29181846
	*MYO6*	• The knockdown inhibits the growth and results in the apoptosis of PCa cells. • PMID: 27431378
	*ILK*	• The inhibition suppresses the activation of *B/Akt* and induces apoptosis of *PTEN*-mutant PCa cells. • PMID: 10716737

*PCa, prostate cancer; PMID, PubMed ID*.

### GO and Pathway Enrichment Analysis

**Figure 4:**
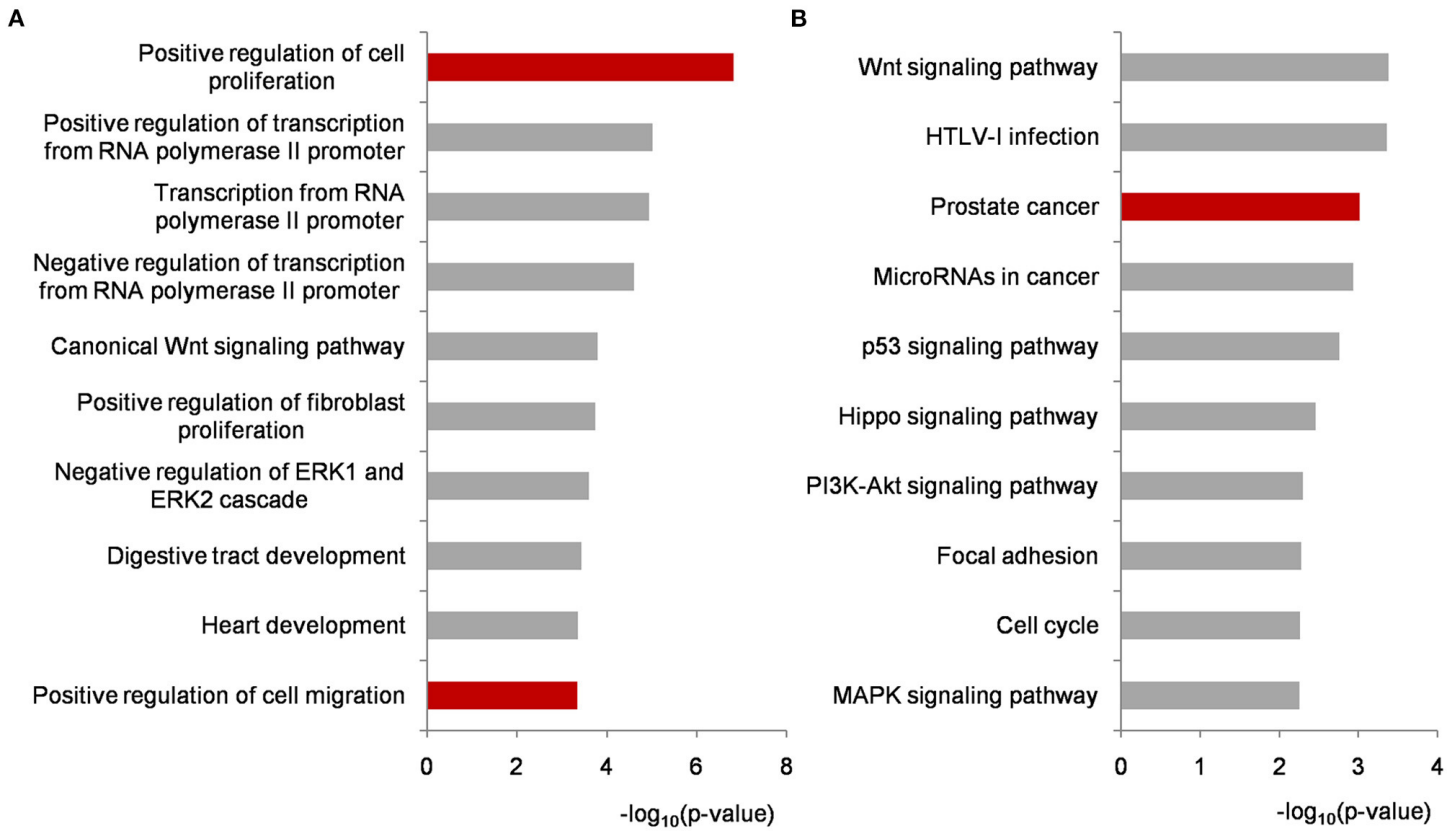
Functional enrichment analyses on biomarker miRNA targets shared by PCa occurrence and progression-specific networks. **(A)** Top ten significantly enriched BP terms. **(B)** Top ten significantly enriched KEGG pathways. All of the *p*-values were negative 10-based log transformed. PCa, prostate cancer; BP, biological process; KEGG, Kyoto Encyclopedia of Genes and Genomes.

Functional enrichment analyses were performed on biomarker miRNA targets shared by PCa condition-specific networks to help decipher the miRNA-PCa carcinogenetic relationships at the GO and pathway level. In terms of the GO analysis, three domains, i.e., biological process (BP), cellular component (CC) and molecular function (MF), were annotated, respectively. As listed in Figure 4A and Supplementary Table 2, some of the significant BP terms were associated with the positive or negative regulation of cell proliferation and migration, indicating the potential role of the identified miRNAs in PCa invasion and metastasis. In the domain of CC, cytoplasm, nucleus, nucleoplasm, cytosol and extracellular exosome were the top-five ranked items and at the MF level, transcriptional activator activity, RNA polymerase II core promoter proximal region sequence-specific binding, protein binding, transmembrane receptor protein tyrosine kinase activity, and transcription factor binding etc. uncovered important clues for molecular function understanding.

To better investigate the mechanisms of the identified miRNAs in PCa, pathway enrichment was conducted and analyzed. As illustrated in Figure 4B and Supplementary Table 2, most of the significantly enriched terms were closely involved in PCa development, such as *Wnt* signaling, prostate cancer, microRNAs in cancer, *p53* signaling, *PI3K-AKT* signaling, and *MAPK* signaling etc. For example, *Wnt* signaling is implicated in PCa-related osteoblast differentiation as a key driver. It could activate *AR*-mediated transcription and promote cell proliferation of androgen-independent PCa (Seo et al., 2017; Wang et al., 2020). The genetic variants or abnormal regulations in *Wnt* signaling provide new approaches for predicting the aggressive behavior of PCa (Shu et al., 2016), and bring candidate targets for PCa personalized therapy (Nandana et al., 2017). Currently, extensive efforts have demonstrated that miRNAs could influence PCa cell activities by regulating genes to inactivate *Wnt* signaling pathway (Du et al., 2019; Ghafouri-Fard et al., 2020). Compared with our previous research, the prostate cancer signaling was enriched higher in this study (Lin et al., 2018a). As shown in Supplementary Figure 1, *GF* and *GFR* controlling the signal transduction from outside to inside of cell membrane were regulated by the identified miRNAs, and the remaining targets were potentially associated with PCa cell proliferation and survival. *p53* is a well-known tumor suppressor protein responding to various cellular stresses such as the growth, invasion and metastasis in PCa development (Takayama et al., 2018; Zhang et al., 2020). As another two cancer-related pathways, *PI3K-AKT* and *MAPK* signaling have been widely reported as targets of miRNAs and genes during PCa activation (Wu et al., 2019; Zheng et al., 2019). In particular, the crosstalk and signaling cascades among *I3K-AKT-mTOR, MAPK, AR*, and *Wnt* improve the mechanistic insights into PCa tumorigenesis and accelerate the understanding in androgen-deprivation therapeutics for precision medicine and personalized healthcare of PCa patients (Shorning et al., 2020).

## Discussion

PCa is developed with increasing incidence and high heterogeneity. In clinical practice, strategies used for PCa screening and monitoring have improved over the years, however, it still lacks sensitive factors to indicate the dynamical changes within prostate signals at the early stage. As a member of non-coding RNAs, miRNAs are reported to regulate gene expression in various biological processes including PCa carcinogenesis, which provide an attractive direction for PCa precision medicine and personalized healthcare.

In the era of translational informatics and intelligent medicine, systems biology creates unprecedented opportunities to integrate multi-dimensional data for computer-aided knowledge discovery. In this study, we collected gene expression and miRNA-mRNA association datasets to identify and explore key miRNAs as candidate biomarkers for PCa holistic management at the network level. Compared with our previous studies solely considering the metastatic or castration-resistant status of PCa (Zhu et al., 2015; Lin et al., 2018a), this study updated the datasets and bioinformatics parameters for model refining, and focused on the functional role of miRNAs associated with the whole development process in PCa, therefore two condition-specific miRNA-mRNA networks were respectively constructed to describe the regulatory pattern and dynamical change between PCa occurrence and progression. In particular, the hub theory and single-line regulation pattern of miRNAs were integrated for the first time to measure the regulatory power, and miRNAs locating at hub sites to independently regulate genes were extracted from each network system based on the definition and characterization of network topologies. Finally, nine miRNAs shared by two networks, i.e., *hsa-miR-1-3p, hsa-miR-125b-5p, hsa-miR-145-5p, hsa-miR-182-5p, hsa-miR-198, hsa-miR-22-3p, hsa-miR-24-3p, hsa-miR-34a-5p*, and *hsa-miR-499a-5p*, were chosen as candidate biomarkers during PCa evolution for performance evaluation and carcinogenic survey.

To validate the potential of the identified miRNAs, ROC and clustering analysis were sequentially performed to test the ability in PCa diagnosis and prognosis. Fortunately, most of these miRNAs achieved higher AUC both in differentiating normal vs. PCa and pPCa vs. mPCa samples based on the prediction and an independent validation dataset, which indicated the predictive power of the miRNAs. Among them, *hsa-miR-145-5p* was top-ranked as the key factor and the result was highly consistent with that in our previous findings using different training datasets and network analysis strategies (Lin et al., 2018a). According to PubMed literature searching, seven of the miRNAs have been reported to be associated with PCa genesis and could serve as biomarkers or therapeutic targets for PCa prevention. Combing with computational prediction, key miRNA-mRNA were screened to decode the relationships between miRNA genotypes and PCa phenotypes at the gene and pathway level, respectively. In particular, *hsa-miR-145-5p/NDRG2/AR* and *hsa-miR-145-5p/KLF5/AR* axis were inferred to be latent mechanisms during PCa occurrence and progression according to bioinformatics identification and literature validation. Moreover, pathways including *Wnt* signaling, prostate cancer, microRNAs in cancer, *p53* signaling, *PI3K-AKT* signaling, and *MAPK* signaling etc. were significantly enriched for pathogenesis understanding.

It should be admitted that several limitations still need to be considered. First, the structural robustness is comprehensively weighted in this study, however, the proposed model lacks sufficient information related to the biological function of miRNAs and mRNAs. Hence the computational framework can be updated by reasonably adding PCa-associated genes as prior knowledge to improve the specificity of miRNAs in PCa carcinogenesis. Second, the complexity and diversity of the background network is not powerful enough. The development of PCa is a dynamical process, so that changeable signals in the network system from normal to different PCa states are meaningful for capturing and comparison. To better understand the heterogeneity across PCa stages, miRNAs specific to PCa conditions, e.g., the occurrence, invasion, metastasis and therapeutic intervention, should be respectively analyzed. Last but the most important, it is difficult to collect enough samples of advanced or mPCa in a short period of time due to the inoperability of these patients. In our future work, low-throughput experiments using wet-lab approaches such as qPCR and western blot will be conducted to validate the identified miRNAs and miRNA-mRNA regulations for biomarker measurement and long-range clinical translation.

## Data Availability Statement

The original contributions presented in the study are included in the article/Supplementary Materials, further inquiries can be directed to the corresponding author/s.

## Author Contributions

BS, YL, and YH designed this study. YL and ZM collected the data. YL and BS proposed the bioinformatics model. YL, ZM, XZ, and XW performed the analysis. YL, JH, YH, and BS wrote and revised the manuscript. BS and YH conceived and supervised the study jointly. All the authors read and approved the final manuscript.

## Conflict of Interest

The authors declare that the research was conducted in the absence of any commercial or financial relationships that could be construed as a potential conflict of interest.
