# Supplementary material for: Identification of Key MicroRNAs and Mechanisms in Prostate Cancer Evolution Based on Biomarker Prioritization Model and Carcinogenic Survey
Source: Front Genet. 2021 Jan 15;11:596826. doi: 10.3389/fgene.2020.596826 (PMC7844321; doi:10.3389/fgene.2020.596826)
Supplement: Supplementary file 1 [file Data_Sheet_1.DOC]

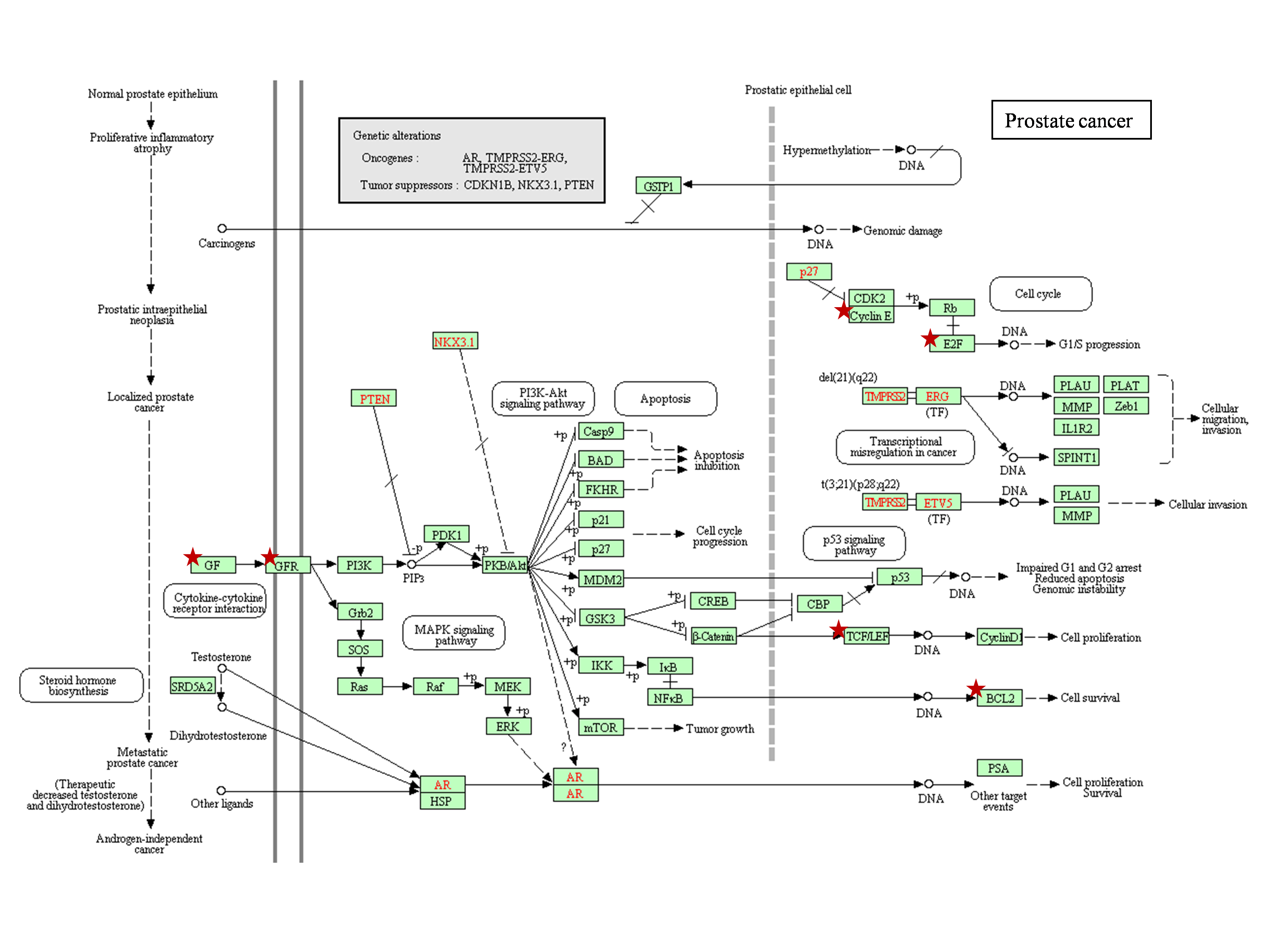


**Figure S1.** The prostate cancer pathway retrieved from DAVID online. The red stars represent enriched targets of biomarker miRNAs. Abbreviation：DAVID: Database for Annotation, Visualization and Integrated Discovery.
